# An Uneven Path Forward: The History of Methylmercury Toxicity Research

**DOI:** 10.1289/ehp.118-a352b

**Published:** 2010-08

**Authors:** Julia R. Barrett

**Affiliations:** **Julia R. Barrett**, MS, ELS, a Madison, WI–based science writer and editor, has written for *EHP* since 1996. She is a member of the National Association of Science Writers and the Board of Editors in the Life Sciences

Organic mercury compounds were first described in the 1800s, with fatal cases of methylmercury poisoning reported in 1865. Early reports described a distinct set of symptoms of methylmercury toxicity, including altered sensation in the face and extremities, tunnel vision, deafness, loss of coordination, and impaired speech. Nearly a century later, against a backdrop of widespread environmental contamination, the clinical picture reappeared, and suspicions of additional harm to human health had developed. Yet it wasn’t until 2009 that international agreement to control mercury pollution was reached. A historical review suggests that—as one early commenter observed—the tunnel vision, forgetfulness, and lack of coordination that symptomize methylmercury toxicity can also affect the conduct and interpretation of environmental health research **[*EHP*** **118(8):1137–1145; Grandjean et al.]**.

Methylmercury became commercially important as a crop fungicide around 1914. Worldwide use was accompanied by worker poisonings and several large-scale food poisoning incidents. The compound emerged as an industrial pollutant in the early 1950s around Japan’s Minamata Bay, where contaminated seafood induced neurologic symptoms mirroring those reported in 1865. Epidemiologic evidence from Minamata, paired with a 1952 report from Sweden, indicated more severe disease from prenatal and early-life exposures, with symptoms including mental retardation, seizures, and impaired motor development. In the 1960s, advances in analytical technology permitted chemical analysis of mercury species in environmental samples, resulting in the discovery of methylmercury biomagnification in the food chain and identification of environmental methylation of inorganic mercury in waterways. Methylmercury had become a worldwide problem, not simply a local issue.

Defining the scope of the problem, much less acting to address it, has involved a political, legal, and ethical maze set on an ever-evolving and still-incomplete scientific foundation. Initially, the inability to identify mercury species in the environment hampered researchers’ efforts to link the presence of methylmercury with poisoning symptoms. That link also was blurred by a time lag of weeks to months between exposure and initial symptoms as well as slow recognition of the significance of experimental and wildlife data. Industrial suppression of toxicity data impaired accurate risk assessment, as did imprecise estimates of exposure, delayed recognition of low-dose effects, and use of adult data only.

Consequently, regulatory safeguards established in the 1960s and 1970s are now known to be inadequate, but improvements have been deferred in light of scientific uncertainties. For example, although it remains unknown whether a “safe” threshold exists for prenatal and early-life exposure to methylmercury, evidence of methylmercury damage to neurodevelopment has been accumulating since the 1950s. More research is certainly needed, the authors write, but prevention and correction of environmental health problems need not and should not be delayed by a desire for absolute proof.

